# Poor quality of sleep and musculoskeletal pains among highly trained and elite athletes in Senegal

**DOI:** 10.1186/s13102-023-00705-4

**Published:** 2024-02-22

**Authors:** Jerson Mekoulou Ndongo, Elysée Claude Bika Lele, Hassane Malam Moussa Ahmet, Wiliam Richard Guessogo, Mbang Biang Wiliam, Jessica Guyot, Vianey Rozand, Ahmadou, Clarisse Noel Ayina Ayina, Loick Pradel Kojom Foko, Nathalie Barth, Bienvenu Bongue, Abdoulaye Ba, Abdoulaye Samb, Fréderic Roche, Samuel Honoré Mandengue, Peguy Brice Assomo-Ndemba

**Affiliations:** 1 Physical Activities and Sport Physiology & Medicine Unit, Faculty of Science, University of Douala, Douala, Cameroon; 2 Faculty of Health Sciences of the Abdou Moumouni University of Niamey, Niamey, Niger; 3 National Institute of Youth and Sports Yaoundé, University of Yaounde I, Yaounde, Cameroon; 4 Mines Saint-Etienne, INSERM, U1059 Sainbiose, Université Jean Monnet, Saint-Étienne, 42023 France; 5 Université Jean Monnet Saint-Etienne, Lyon 1, Université Savoie Mont-Blanc, Laboratoire Inter-universitaire de Biologie de la Motricité, F-42023 Saint-Étienne, France; 6 Support and Education Technical Centre of Health Examination Centres (CETAF), Saint-Etienne, France; 7 Faculty of Medicine, Pharmacy and Dentistry, University of Cheikh Anta Diop, Dakar, Sénégal; 8 Faculty of Medicine and Biomedical Science, University of Yaounde I, Yaounde, Cameroon

**Keywords:** Poor sleep quality, Musculoskeletal pains, Highly trained and elite athletes, Senegal

## Abstract

**Background:**

Previous studies reported that poor sleep quality (PSQ) was associated with musculoskeletal pains (MSP) and poor physical performance in athletes.

**Objective:**

The current study aimed at determining PSQ and its associations with MSP in some sub-Saharan athletes.

**Methods:**

A cross sectional study was conducted among 205 highly trained and 115 elite athletes (aged: 25 ± 2 years, Body mass index: 22.8 ± 0.9 kg/m^2^) in Dakar, Senegal, during a competitive season in a variety of sport disciplines including athletics, basketball, football, rugby, wrestling, tennis. Quality of sleep and MSP were assessed using the French version Pittsburgh Sleep Quality Index (PSQI) and French version of Nordic questionnaire respectively. Pain on body joints during a week was defined as seven-day MSP (MSP-7d) and PSQ for a PSQI > 5.

**Results:**

27.8% (95%CI: 23.2–32.9) of the overall sample suffered PSQ, with 33.7% (95%CI: 24.7–44.0) in basketball and 24.7% (95%CI: 16.9–34.6) in football. According to athletic status and gender, PSQ was more prevalent among highly trained (66.3; 95%CI: 55.9–75.3) and men (69.7%; 95%CI: 59.5–78.7). Among athletes with PSQ 43.8% (95%CI: 33.9–54.2) suffered MSP-7d, with 36.6%; highly trained (95%CI: 23.7–42.9) and 28.1% female. Considering body region, hips/thigh (14.6%; 95% CI: 8.74–23.4) and upper back (13.5%; 95%CI: 7.88 -21, 1) were more affected. Basketball players were more affected from MSP (MSP-7d = 38.5%; 95%CI: 24. 9–54.1) on high on wrists/hands (MSP-7d = 44.4%; 95%CI: 18.9 -73.3; *P* = *0.04*). Based on athletic status, MSP-7d were higher on highly trained necks (100%; 95%CI: 56.1–100; *p* = *0.04*). PSQ was associated with basketball (OR: 3.062, 95%CI: 1.130–8.300, *p* = *0.02*) compared to Athletic. PSQ and MSP-7d were associated on Wrist/hands (OR: 3.352, 95%CI: 1.235–9.099, *p* = *0.01*), and at the upper back (OR: 5.820, 95%CI: 2.096–16.161, *p* = *0.0007*).

**Conclusion:**

These results indicate that PSQ is considerable among Senegalese athletes and is associated with MSP during a week. Hence, we recommend to look for strategies optimizing good quality of sleep in order to reduce pains, to improve health.

## Background

Sport both in developed and developing countries requires a follow-up (physiological, psychological and nutritional) of the major actors who are the athletes for health preservation and the optimization of biomechanical [1, 2]. This follow-up during training session or competition also allows for assessing performances, profiling athletic talent, identifying ability to compete, and identifying weakness and factors determining physical performance [3]. The follow-up of the athlete emphasizes the notion of recovery by the sleep amply studied [4, 5].

Sleep is a physiological condition characterized by a reversible behavioral state, with changes in the level of consciousness and responsiveness to stimuli. Sleep is a fundamental requirement for health and recovery that would be actively involved in homeostatic processes able to revitalize and restore the main physiological, metabolic and psychological functions. This condition is necessary for physiological mechanisms essential to life, such as the energy restoration, neural plasticity and secretion of the growth hormone [6]. Persistent PSQ has a cumulative long-term negative effect on human health outcomes, resulting in increased susceptibility to infections [7], and non-communicate diseases like hypertension, dyslipidemia, cardiovascular disease, weight-related issues, metabolic syndrome, type 2 diabetes mellitus, and colorectal cancer [8]. Sleep deprivation studies report a significant association with the decreased of cognitive function [9], decreased mood [10].

Sleep disturbance is known to exert detrimental effects on physiological and physical performance of athletes and deteriorate health [11, 12]. PSQ has been associated with musculoskeletal pains (MSP) and reported to aggravate it, and causes injuries [13]. MSP are frequently observed in athletes and can often be the cause of poor performances and even loss of competition. MSP are a common problem and important warning signals of overuse injury in athletes [14, 15]. Injuries in athletes most of the time require significant recovery periods with physiotherapy or surgery intervention [16, 17]. Prevention of MSPs and injuries in sports medicine constitute one of the most worrying health problems because resulting in high economic costs, withdrawal of athletes from training and competition, and impairment of their performance [18, 19]. It is therefore appropriate to prevent the occurrence of MSP in athletes by promoting good sleep. Moreover, poor sleep and pain are two interdependent and bidirectional phenomena drawing a vicious circle that can disrupt athlete’s performance and health. Pain can interrupt or disrupt sleep, just as changes in sleep patterns can also influence pain perception [13, 20].

In most sub-Saharan Africa developing countries, sport is a unifying tool, requiring appropriate scientific interventions for performance optimization due to better follow-up of physiological adaptations related to the restorative aspect of sleep. However, not many information’s are known on MSP in African athletes. Few studies have been published on MSP [21, 22] our knowledge, none coupling sleep quality and MSP. Hence, the purpose of the present study was to evaluate sleep quality in highly trained and elite athletes in Senegal and to investigate possible associations between sleep quality and MSP.

## Methods

### Study design and participants

This was a cross-sectional prospective and analytical study was conducted in Dakar, Senegal during four months. After obtaining authorizations of the administrative and technical staff following the explanation of the purpose of the study and the potential benefits on health and performance of the athletes; participants were recruited from football, basket-ball, rugby, tennis, athletics, and traditional wrestling professional and amateur clubs. Athletes were interviewed by the investigators who collected the data by completing the questionnaires. Athletic status (highly trained and elite) of participants has been characterized according to McKay et al. [23] classification. We excluded athletes who take caffeine or energy drink in the evening and those recovering from musculoskeletal trauma, under rehabilitation or physiotherapy interventions.

### Sampling

The sample size was determined by using the Lorentz’s formula [N = p (1-p) z^2^/d^2^ where N is the minimum sample size; p: prevalence; z = 1.96 for confidence at 95%; d = 0.05] with the prevalence of PSQ of *85.71%* reported in a month among athletes in Canadian athletes [24]. Then, the minimal sample size found was 188 participants. A total of 320 athletes was finally included.

### Ethics

After explaining the aim and specifics objectives of the study to administration staff of athlete clubs and coaches; administrative authorization and the purpose of the research was also explained to athlete. Thereafter an informal written and signed consent was obtained from each participant with the possibility to withdraw from the study at any time. The national ethics committee of Cheikh Anta Diop University of Dakar, Senegal approved (015/2021/CER/UCAD) the study which was conducted in accordance with the recommendations of the Declaration of Helsinki revised in 1989.

#### Data collection

##### Socio-professional information’s and anthropometric measures

A structured questionnaire was elaborated to collect personal information of athletes such as age, gender, case of injuries, athletic status, sport discipline, number of training session/ week. The weight was measured using an electronic scale Tanita BC-532 (Tokyo, Japan) and the height a rod graduated to the nearest centimeter. Body mass index (BMI) was calculated using the Quetelet’s formula: BMI (Kg.m^−2^) = Weight (kg) / height^2^ (m^2^).

##### Quality of sleep

A French version of the Pittsburgh Sleep Quality Index (PSQI) was used to assess the sleep quality [25]. This questionnaire assesses the quality of sleep of an individual for a month*.* PSQI consists in 19 items of the following seven components of sleep quality: sleep onset latency, sleep duration, efficiency, quality, disturbances, medication, and daytime dysfunction. Each component of PSQI is scored between 0–3, and the sum of the seven components yields a global score of sleep quality with a total score ranging from 0- 21 points; a high score (PSQI score > 5) is an indication of PSQ.

#### Musculoskeletal pains

A structured validated French version of the Nordic questionnaire adapted to adolescent population was used to determine MSP prevalence [26]. This questionnaire determines the occurrence of MSP on nine body regions (neck, shoulders, elbows, wrists/hands, upper back, lower back, hips/thighs, knees, ankles/feet) during a week. For each body region, the parameters evaluated were:

- Presence or absence of aches, pains or genes during the last seven days,

was used to determine MSP prevalence. This questionnaire determines the occurrence of MSP on nine body regions (neck, shoulders, elbows, wrists/hands, upper back, lower back, hips/thighs, knees, ankles/feet) during a week. For each body region, the parameters evaluated were:

- Presence or absence of aches, pains or genes during the last seven days,
- Bad performance due to joint pains,
- Absenteeism of training session or a competition during the last seven days for reasons related to pain in one or several body regions,
- Presence or not of a history of trauma in one or several body regions during a training session or competition*.*

Therefore:

- A week/7-day MSP (MSP-7d): was defined as seven-day prevalence of MSP. Pain related to a former injuries regions was not considered as MSP.

### Statistical analysis

The data were inserted in the software Microsoft Excel 2016. The analysis was conducted using StatView 5.0 (SAS Institute, Inc., Chicago, USA) software. The Kolmogorov–Smirnov test verified the normality of data. In the descriptive stage of the analysis, mean and standard deviation of numerical variables were calculated, as well as the absolute and relative frequencies of categorical variables and lower and upper limits of the 95% confidence interval for a proportion confidence intervals of MSP-7d and PSQ were determined [27]*.* Unpaired Student’s *t*-test was used to compare unpaired quantitative variables. Besides, the chi-square test was conducted to verify the difference between proportions, and also to examine the association between nominal variables. Logistic regression models were performed to identify factors associated with MSP and PSQ. Then, association was quantified through computing crude odd ratio adjusted on gender, age and number of training sessions per week, at 95% of confidence interval (95%CI). The statistical significance threshold was set for any value of *P* < 0.05.

## Results

Table 1 shows anthropometric parameters, training charge, PSQI, athletic status (highly trained and elite) and sport discipline of participants, comparing between and females.

**Table 1:** Gender, socio-demographic and anthropometric characterizations

	**Categories**	**Total** **(Mean ± SD)**	**Female** **(Mean ± SD)**	**Male** **(Mean ± SD)**	***P*-value α**	**Professionals** **(Mean ± SD)**	**Highly trained** **(Mean ± SD)**	***P*-value β**
**Anthropometric**	Age (years)	25 ± 2	26 ± 1	25 ± 2	< 0.0001	26 ± 4	25 ± 3	0.14
	Height (m)	179 ± 7.6	167 ± 4.5	182 ± 4.2	< 0.0001	1.79 ± 0.90	1.77 ± 0.88	0.03
	Weight (Kg)	73.2 ± 8.5	67.3 ± 8.3	76.1 ± 13	< 0.0001	74.8 ± 12.8	72.6 ± 12.1	0.13
	BMI (Kg/m^2^)	22.8 ± 0.9	21.2 ± 2	23.2 ± 0.1	0.9	23.3 ± 4.1	23.9 ± 10.8	0.51
**Training sessions**	Number/week	4 ± 1	3 ± 1	4 ± 1	< 0.0001	5.4 ± 1.2	3.9 ± 1.4	< 0.0001
**PSQI components**	Sleep onset latency	1.02 ± 0.55	0.99 ± 0.53	1.03 ± 0.56	0.5	0.99 ± 0.50	1.03 ± 0.58	0.55
	Sleep duration	1.05 ± 0.82	1.12 ± 0.85	1.02 ± 0.80	0.3	1.13 ± 0.86	1.01 ± 0.79	0.2
	Sleep efficiency	0.45 ± 0.55	0.50 ± 0.54	0.39 ± 0.56	0.1	0.39 ± 0.54	0.44 ± 0.56	0.4
	Sleep quality	0.06 ± 0.27	0.06 ± 0.24	0.06 ± 0.28	0.6	0.10 ± 0.32	0.04 ± 0.22	0.09
	Disturbances	0.98 ± 0.62	1.03 ± 0.57	0.95 ± 0.64	0.2	0.96 ± 0.54	0.99 ± 0.66	0.68
	Medication	0.31 ± 06.4	0.31 ± 0.68	0.31 ± 0.62	0.9	0.25 ± 0.58	0.35 ± 0.67	0.2
	Daytime dysfunction	0.83 ± 0.77	0.70 ± 0.72	0.89 ± 0.79	0.04	0.86 ± 0.77	0.78 ± 0.78	0.3
	**PSQI Index**	4.67 ± 1.69	4.63 ± 1.67	4.68 ± 1.70	0.7	4.60 ± 1.65	4.70 ± 1.72	0.6
		n(%)	n(%)	n(%)	*P*-valueα			
**Athletic status**	Elite	115(35.9)	30(26.1)	85(73.9)	0.21			
	High trained	205(64.1)	67(32.7)	138(67.3)		n(%)	n(%)	*P*-valueβ
**Disciplines**	Athletics	42(13.1)	19(45.2)	23(54.8)	< 0.0001	21(50)	21(50)	< 0.0001
	Basket-ball	80(25)	38(47.5)	42(52.5)		39(48.8)	41(51.3)	
	Football	90 (28.1)	0(0.0)	90(100)		41(45.6)	49(54.4)	
	Rugby	58(18.1)	28(48.3)	30(51.7)		0(0.0)	58(100)	
	Wrestling	14 (4.4)	0(0.0)	14(100)		14(100)	0(0.0)	
	Tennis	36(11.3)	12(33.3)	24(66.7)		0(0.0)	36(100)	

*PSQI* Pittsburgh sleep quality index

*P*-value α: gender comparison, *P*-value β: Athletic status comparison

**Fig. 1:**
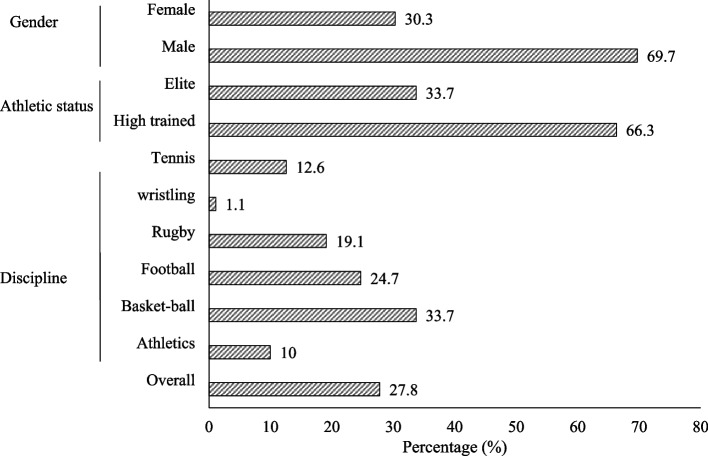
Prevalence of poor PSQ

Prevalence of poor sleep reported among athletes was 27.8% (95%CI: 23.2–32.9), and was more prevalent in basketball (33.7%; 95%CI: 24.7–44.0) followed by football (24.7%; 95%CI: 16.9–34.6) and rugby (19.1%; 95%CI: 12.3–28.5). Furthermore, according to athletic status and gender, PSQ was more prevalent among highly trained (66.3; 95%CI: 55.9–75.3) and men (69.7%; 95%CI: 59.5–78.7) (Fig. 1).

**Fig. 2:**
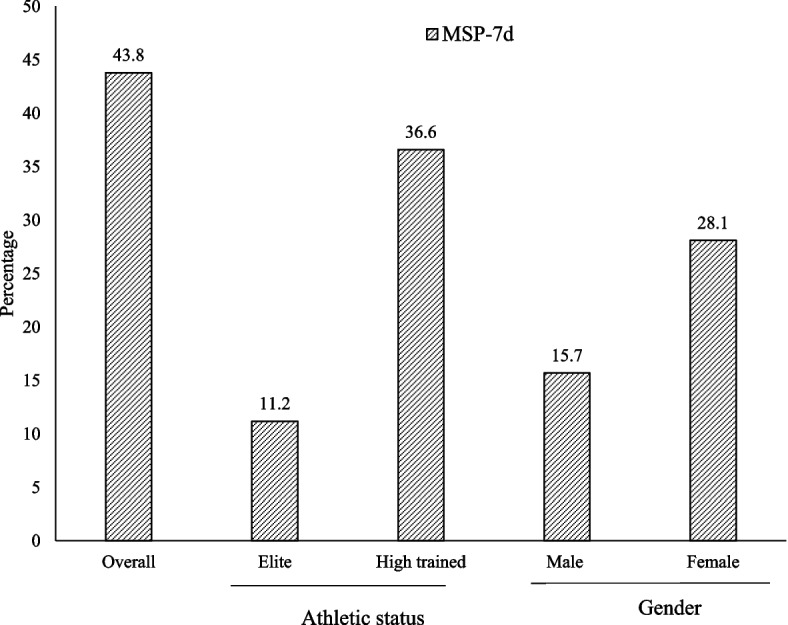
MSP in athletes with PSQ according to gender and athletic status. MSP-7d: Prevalence of musculoskeletal pains during the seven last days

Among, athletes having PSQ 43.8% (95%CI: 33, 9–54, 2) were suffering from MSP during the last seven days, with no significant difference between gender and athletic status (Fig. 2).

**Fig. 3:**
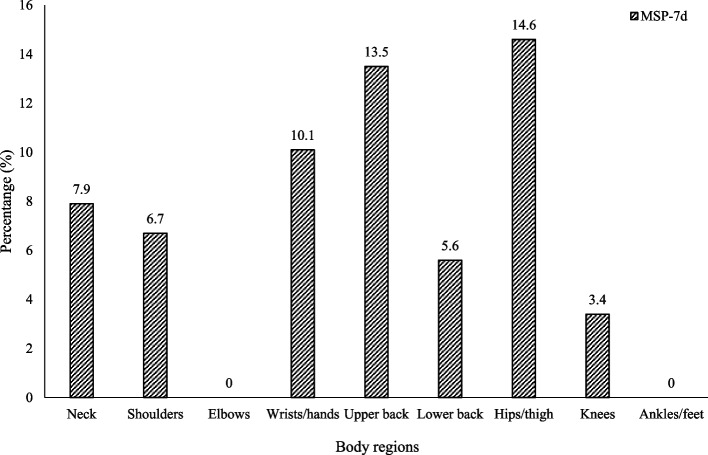
Prevalence of MSP according to body regions in athletes with PSQ MSP-7d: Prevalence of musculoskeletal pains during the seven last days

MSP-7d were more located on hips/thigh (14.6%; 95% CI: 8, 74–23, 4) followed by upper back (13.5%; 95%CI: 7, 88–21, 1) and Wrists/hands (10.1%, 95%CI: 5, 01–18, 1) in participants with poor sleep (Fig. 3).

In athletes with poor sleep, during the last 7 days, basketball had significant prevalence of MSP (MSP-7d = 38.5%; 95%CI: 24. 9–54.1; *P* = *0.04*) and high on wrists/hands (MSP-7d = 44.4%; 95%CI: 18.9–73.3; *P* = *0.04*). Based on athletic status, MSP were higher on highly trained necks (100%; 95%CI: 56.1–100; *P* = *0.04*) (Table 2).

**Table 2:** Prevalence of MSP in participants with bad quality of sleep according to body region, sport discipline and athletic status

**Discipline**	**MSP- 7d ( +)**	**Neck**	**Shoulders**	**Elbows**	**Wrists/** **hands**	**Upper** **back**	**Lower** **back**	**Hips/** **thigh**	**Knees**	**Ankles/** **feet**
Athletics	4(10.3)	0(0.0)	1(16.7)	0(0.0)	1(11.1)	1(8.3)	0(0.0)	2(15.4)	1(33.3)	0(0.0)
Basket-ball	**15(38.5)**	4(57.1)	3(50.0)	0(0.0)	**4(44.4)**	3(25.0)	2(40.0)	6(46.2)	2(66.7)	0(0.0)
Football	4(10.3)	1(14.3)	0(0.0)	0(0.0)	2(22.3)	1(8.3)	0(0.0)	1(7.7)	0(0.0)	0(0.0)
Rugby	6(15.4)	2(28.6)	0(0.0)	0(0.0)	0(0.0)	4(33.3)	1(20.0)	0(0.0)	0(0.0)	0(0.0)
Wristling	1(2.6)	0(0.0)	0(0.0)	0(0.0)	1(11.1)	0(0.0)	0(0.0)	0(0.0)	0(0.0)	0(0.0)
Tennis	9(23.1)	0(0.0)	2(33.3)	0(0.0)	1(11.1)	3(25.0)	2(40.0)	4(30.8)	0(0.0)	0(0.0)
*P*-value α	**0.01**	0.06	0.29	NA	**0.04**	0.3	0.39	0.06	0.4	NA
**Status**										
Elite	10(25.6)	0(0.0)	2(33.3)	0(0.0)	5(66.7)	3(25.0)	1(20.0)	4(30.8)	1(25.0)	0(0.0)
High trained	29(74.4)	**7(100.0)**	4(66.7)	0(0.0)	3(33.7)	9(75.0)	4(80.0)	9(69.2)	2(75.0)	0(0.0)
* P*-value β	0.15	**0.04**	0.9	NA	0.9	0.4	0.5	0.8	0.9	NA

*MSP-7d* Prevalence of musculoskeletal pains during the seven last days, *NA* Not available

*P*-value α: comparison of MSP-7d according to body regions in sport discipline

*P*-value β: comparison of MSP-7d according to athletic status on body regions

PSQ was associated with sport discipline, basketball players were more at risk (OR: 3.062, 95%CI: 1.130–8.300, *P* = 0.02) compared to Athletic. Athletes with PSQ had almost nearly 2 time the risk of MSP-7d (OR: 1.941, 95%CI: .171 -3.235, *P* = *0.02*), according to body region this risk was present on neck (OR: 3.132, 95%CI: 1.014 – 9.806, *P* = 0.04), Wrist/hands (OR: 3.352, 95%CI: 1.235–9.099, *P* = 0.01), and at the upper back (OR: 5.820, 95%CI: 2.096–16.161, *P* = 0.0007) (Table 3).

**Table 3:** Risk factor of poor sleep

**Categories**	**Sport disciplines**			**MSP-7d**							
	Athletic	Basketball		Total		Neck		Wrist/hands		Upper back	
	OR (95%CI) *P*-value	Crude OR (95%CI) *P*-value	Adjusted OR (95%CI) *P*-value	Crude OR (95%CI) *P*-value	Adjusted OR (95%CI) *P*-value	Crude OR (95%CI) *P*-value	Adjusted OR (95%CI) *P*-value	Crude OR (95%CI) *P*-value	Adjusted OR (95%CI) *P*-value	Crude OR (95%CI) *P*-value	Adjusted OR (95%CI) *P*-value
Pood sleep	1	1.985 (0.506–7.792) **0. 32**	3.062 (1.130–8.300) **0.02**	1.950 (1.175–3.237) **0.009**	**1.941** (1.171 -3.235) **0.01**	**3.509** (1.014 – 9.806) **0.04**	**3.132** (1.019 – 9.626) **0.04**	3.136 (1.170–8.407) **0.02**	3.352 (1.235–9.099) **0.01**	5.844 (2.121–16.105) **0.0006**	5.820 (2.096–16.161) **0.0007**

*MSP-7d* Prevalence of musculoskeletal pains during the seven last days, *OR* Odd ratio, *CI* Confidence interval

## Discussion

The present study aimed at determining prevalence of PSQ in athletes, and then its association with MSP in a week period. Prevalence of poor sleep reported among athletes was 27.8%. This prevalence is similar to that reported by Gomes et al. [28] among adolescent amateur athletes in five sport disciplines (volleyball, handball, basketball, swimming and judo). Some studies highlighted that PSQ is generally high in athletes, such as noticed in the sample of the study of Samuels [24] in Canadian athletes who observed an important worse poor sleep of 85.7%. A study of Juliff et al. [29] in a sample of 283 elite Australian athletes reported important prevalence of PSQ of 64.0%. and Silva et al. [30] in a study among 146 athletes from the Brazilian Olympic team found 53% of sleep complaints. In a recent study between elite and sub elite athletes from a wide variety of sports Madigan et al. [31] in a reported an important rate of poor sleep of 64% and 65% respectively. All these results suggest that PSQ is a crucial problem among athlete requiring education of sleep for good health and best performance.

According to athletic status, no statistical difference of poor sleep between amateur and professional athletes has been noticed. This result is in accordance to that reported by Madigan et al. [31] in a study between elite and sub elite athletes without difference of poor sleep between both groups. However, in a recent study of Penttilä et al. [32] it was noticed an important proportion of poor sleep in professional athletes compare to amateur. With regards to physiological and psychological complexity of sleep, the poor quality of sleep should be superior in professional athletes due to the important mental stress and physical demands. Moreover, it is well established that training as found significantly superior in professional athletes compared to amateur in the present study constitutes an important determinant of sleep deficiency difference [10, 33, 34].

According to sport discipline, poor sleep was more prevalent in basketball. This result is similar to that observed by Franco et al. [35] in a study on quality of sleep in athletes involved four sport (athletics, boxing, basketball and crossfit) poor sleep was mostly in athletes practicing crossfit and basketball.

With respect to gender, no significant difference on poor quality of sleep was noticed. This result is in accordance to that of Juliff et al. [29] who didn’t found gender difference of sleep disorders in Australian athletes. Gender differences of sleep disorders are very controversies. Brand et al. [36] in a pilot study on self-reported sleep quantity and sleep-related personality traits in adolescents found that female adolescents were at greater risk of suffering of poor sleep; of the same with Schaal et al. [37] who observed highest rate of sleep disorders in women. Franco et al. [35] who analyzed sleep in 83 athletes, in individual and collective sports found that the majority of bad sleepers were women. Nevertheless Silva et al. [30] in the clinical evaluation reveal that men reporting more sleep complaints than women elite athletes. Previous studies argued that women’s sleep patterns are subject to disturbance because they are determined by physiological factors like changes in hormonal variation, social roles and interactions [36–38].

Among athletes with poor sleep 43.8% were suffering from MSP during the last seven days without significant difference according to gender and athletic status. MSP-7d were more on hips/thigh followed by upper back and wrists/hands. Studies assessing quality of sleep and the occurrence of MSP in athletes are scarce. However, de Souza Bleyer et al. [39] investigating associations between sleep quality and the occurrence of musculoskeletal complaints among elite athletes in Brazil found MSP during the last seven days on six body region excepted neck, shoulders and hip/thighs in athletes with poor sleep. Moreover, this lack of difference in pain between genders does not corroborate the study of Shan et al. [40] suggesting that beyond the fact that poor quality of sleep should be predominant in women, pain should be more in females because of their higher pain perception threshold than males. Also, Anatomical characteristics of the female body have been implicated as enhancing the development of pain in some body regions such as the back and therefore leading to a higher prevalence in women than in men [41]. In addition, pain in women may be related to their lower muscle mass and bone density, which may lead to destabilization of the body and therefore insufficient compensation for high loads [42].

In the present study it was noticed in athletes with poor sleep during the last 7 days, high MSP-7d in basketball. Also, poor sleep was associated with sport discipline. Basketball players with poor sleep were in risk to suffer from MSP in a week compared to Athletic. In a recent study on MSP in amateur and professional athletes, Malam et al. [22] found high rate of MSP-7d basketball players. Added to their PSQ which can justify high prevalence of pain, basketball requiring high physiological and biomechanics demands in aerobic and anaerobic capacities along with integration of physical characteristics. Also, frequent jumping, landing and changes in direction make up much of physical load of competitive games, which therefore, expose basketball players to high level of eccentric muscle contractions and joints solicitations which can be causes of pain [43].

Many researchers had established a link between, sleep quality and pain, thus establishing a vicious circle [13, 44, 45]. Based on physiological justifications, poor sleep is accompanied by an increased sensitivity to noxious stimuli and a decrease in endogenous pain-inhibitory [46–48]. Others possible neurophysiological mechanisms connecting sleep to pain are focusing on ghrelin [49, 50].

According to Guneli & Ates [50], beyond the control of food intake ghrelin secreted primarily by the stomach, links to its hypothalamic receptor at the arcuate nucleus. It’s shown that sleep is related to ghrelin [51] with a rise levels at night [52, 53] its level rises primarily in response to acute sleep disturbance [54]. Then ghrelin directly activates the neuropeptide Y and indirectly inhibits proopiomelanocortin neurons in the hypothalamus. Activated neuropeptide Y is known to modulate nociception in some regions of the central nervous system, inducing spinal antinociception and regulating pain in the brain. In addition, proopiomelanocortin derivative, β-endorphin, is known as an important endogenous key-component of the antinociceptive system. Then, the antinociceptive effect is mediated by opioid receptors which is modulated by nitric oxide [55]. Ghrelin increasing nitric oxide synthesis levels, may also improve antinociceptive effects of endogenous opioids, showing its interaction with central opioid mechanisms. All the same, In addition to analgesic activity, ghrelin has been indicated to be a powerful anti-inflammatory intermediate and inhibits pro-inflammatory cytokines such as IL-1β, IL-6, and TNF-α, which cause pain and other symptoms [56, 57].

## Conclusion

This study provides evidence of the reality of PSQ in Senegalese athletes, in whom musculoskeletal pain were found. MSP-7d in athletes with PSQ were higher in highly trained and women. The most affected body regions were the hips/thighs and upper back. Thus, the optimization of the biomechanical performances and health of athletes is linked to a good physiological recovery. Recovery is linked to good quality sleep which will reduce the occurrence of MSP. Therefore, it is imperative for sports coaches to emphasize good sleep education in athletes.

## Data Availability

Data can be shared upon contact with the correspondence author.

## Abbreviations

PSQ: Poor sleep quality
MSP: Musculoskeletal pain
MSP-7d: Musculoskeletal pain during the 7 last days
PSQI: Pittsburgh Sleep Quality Index
